# Exosome derived from tumor-associated macrophages: biogenesis, functions, and therapeutic implications in human cancers

**DOI:** 10.1186/s40364-023-00538-w

**Published:** 2023-11-19

**Authors:** Manli Zhou, Xiaoyun He, Cheng Mei, Chunlin Ou

**Affiliations:** 1 Department of Pathology, Xiangya Hospital, Central South University, Changsha, 410008 Hunan China; 2 Departments of Ultrasound Imaging, Xiangya Hospital, Central South University, Changsha, 410008 Hunan China; 3 Department of Blood Transfusion, Xiangya Hospital, Clinical Transfusion Research Center, Central South University, Changsha, 410008 Hunan China; 4 National Clinical Research Center for Geriatric Disorders, Xiangya Hospital, Central South University, Changsha, China

**Keywords:** Tumor-associated macrophages, Exosomes, Tumor microenvironment, Metabolic reprogramming, Liquid biopsy, Therapeutic target

## Abstract

Tumor-associated macrophages (TAMs), one of the most abundant immune cell types in the tumor microenvironment (TME), account for approximately 50% of the local hematopoietic cells. TAMs play an important role in tumorigenesis and tumor development through crosstalk between various immune cells and cytokines in the TME. Exosomes are small extracellular vesicles with a diameter of 50–150 nm, that can transfer biological information (e.g., proteins, nucleic acids, and lipids) from secretory cells to recipient cells through the circulatory system, thereby influencing the progression of various human diseases, including cancer. Recent studies have suggested that TAMs-derived exosomes play crucial roles in malignant cell proliferation, invasion, metastasis, angiogenesis, immune responses, drug resistance, and tumor metabolic reprogramming. TAMs-derived exosomes have the potential to be targeted for tumor therapy. In addition, the abnormal expression of non-coding RNAs and proteins in TAMs-derived exosomes is closely related to the clinicopathological features of patients with cancer, and these exosomes are expected to become new liquid biopsy markers for the early diagnosis, prognosis, and monitoring of tumors. In this review, we explored the role of TAMs-derived exosomes in tumorigenesis to provide new diagnostic biomarkers and therapeutic targets for cancer prevention.

## Background

With the continual development of single-cell sequencing and spatial transcriptome technologies, an increasing number of tumor-type ecosystems have been studied [1, 2]. The tumor microenvironment (TME) is the core aspect of the tumor ecosystem and the location for tumor cell interaction with other tumor and host cells [3, 4]. The TME is composed of immune cells, stromal cells, blood vessels, and the extracellular matrix. As one of the most abundant immune cell types in the TME, tumor-associated macrophages (TAMs) account for approximately 50% of the local hematopoietic cells. TAMs secrete various biological factors, such as inflammatory cytokines, chemokines, growth factors, and exosomes, and they influence cancer cell growth, metabolism, drug resistance, and immune response [5].

Exosomes were discovered by Trams et al. in 1981 [6]. They are extracellular vesicles (EVs), approximately 50–150 nm in diameter, and are present in almost all types of body fluids, such as saliva, urine, and plasma [7]. Exosomes were initially considered cellular waste; however, with expanding research and the popularization of electron microscopy, their biological functions and effects have been revealed. Exosomes regulate intercellular communication by transferring multiple types of cargo, such as RNA, DNA, proteins, and metabolites, to recipient cells, and they participate in different processes of tumor progression [8–10]. In recent years, exosomes have become potential targets for disease diagnosis and management because of their widespread presence and accessibility, providing broad prospects for the development of precision medicine. Through exosomes, TAMs regulate the tumorigenesis through crosstalk between various immune cells in the TME [11]. TAMs-derived exosomes carry non-coding RNAs (ncRNAs), proteins, and lipids that regulate malignant cell proliferation, invasion, metastasis, angiogenesis, immune response, drug resistance, and metabolic reprogramming.

In this article, we present a systematic literature review and analysis of the role of TAMs-derived exosomes in tumorigenesis. We summarize how TAMs-derived exosomes regulate interactions between tumor cells and other cells in the TME, as well as the relationship between TAMs-derived exosomes and the clinicopathological features of malignancies. In addition, we discuss the potential of TAMs-derived exosomes as diagnostic biomarkers and therapeutic targets for cancer, as well as the relevant challenges.

## Macrophages

Macrophages were first discovered by the biologist Ellie Metchnikoff in 1882 while studying starfish larvae. Metchnikoff named these cells phagocytes based on the Greek words *phagein* (phagocytosis) and *kytos* (cells) [12]. Macrophages are white blood cells normally located in tissues, and are derived from monocytes, which in turn are derived from precursor cells in the bone marrow. Macrophages in the brain, skin, lung, heart, liver, and kidney originate from embryonic precursors in the yolk sac and fetal liver and are independent of circulating monocytes during local self-renewal [13]. Different tissue-specific macrophage populations have different transcriptional profiles and epigenetic markers, which are determined by tissue-specific factors [14]. Macrophages have multiple functions, including the recognition and clearance of pathogens, killing of target cells, antigen presentation, and immune regulation. They can rapidly sense and integrate multiple signals from the surrounding environment, thereby participating in homeostasis.

**Fig. 1:**
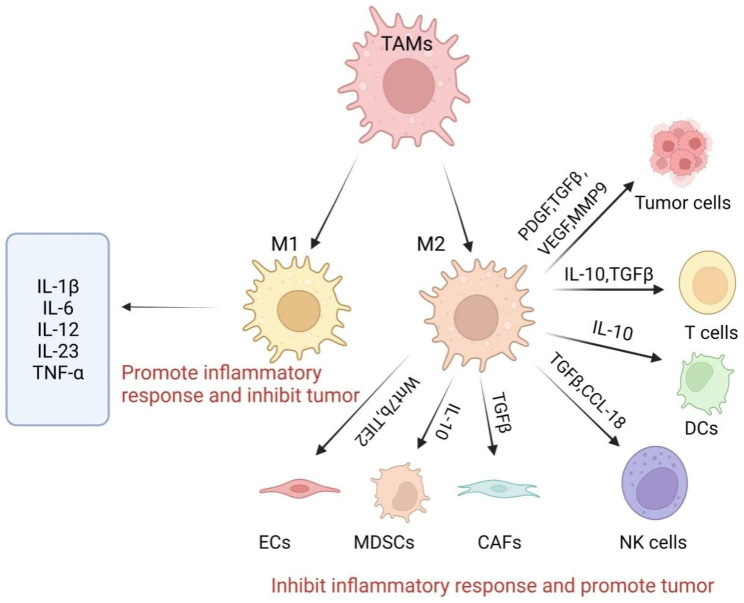
Effects of TAMs on other tumor cells and immune cells in the TME. TAMs secrete a variety of cytokines that facilitate crosstalk with other cell types in the TME to influence tumor progression

Depending on their activation status, function, and secreted cytokines, macrophages can be categorized as one of two subtypes: M1 (pro-inflammatory, classically activated macrophages) and M2 (anti-inflammatory, alternatively activated macrophages) [15, 16]. TAMs have an M1 phenotype in early-stage cancer, and M1-type macrophages express high levels of CD40, CD80, and CD86, which influence antitumor immunity. M1-type macrophages produce pro-inflammatory cytokines, such as interleukin (IL)-1β, IL-6, IL-12, IL-23, and tumor necrosis factor (TNF)-β, which promote inflammatory responses and inhibit tumor growth. They are then stimulated by Th2 cytokines (e.g., IL-4 and IL-13) to polarize at the tumor site and are then activated into M2-type macrophages that produce anti-inflammatory cytokines, such as IL-10 and TGF-β [17]. M2-type macrophages express high levels of CD206, CD163, and TGFβR, which mainly inhibit the inflammatory response, thus promoting tumor growth and metastasis. They also affect other cells in the TME, including cancer-associated fibroblasts (CAFs), endothelial cells (ECs), dendritic cells (DCs), natural killer cells (NK cells), and myeloid-derived suppressor cells (MDSCs) [18]. Normally, M1-M2 polarization is highly dynamic and reversible. Macrophages in the TME, designated as TAMs and predominantly M2 macrophages, are important immune cells in the TME and are essential for tumor development [19]. TAMs secrete a variety of cytokines and inflammatory factors that facilitate crosstalk with other cell types in the TME [20], thereby promoting tumor metastasis, angiogenesis, and immune escape (Fig. 1).

## Exosomes

**Fig. 2:**
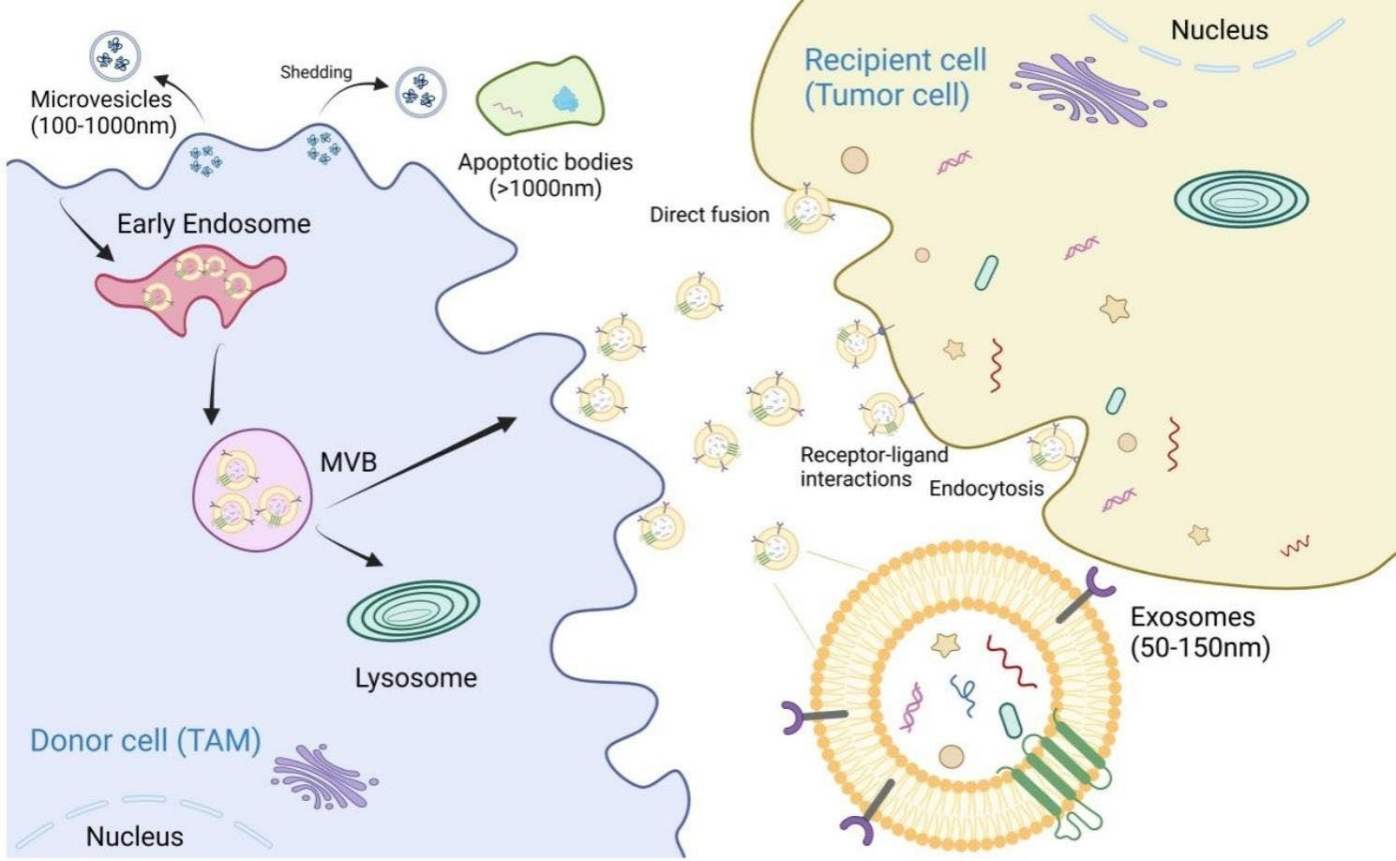
Extracellular vesicle biogenesis and secretion in donor cells and communication with recipient cells. Exosomes participate in intercellular communication by transferring different molecules, including exosomal mRNA, circRNA, lncRNA, miRNA, lipids, and proteins. Exosomes act on neighboring or distant target cell receptors through multiple pathways, including direct fusion, receptor-ligand interaction, and endocytosis

EVs are a class of vesicles with a double-layered phospholipid membrane secreted by cells. They carry a variety of bioactive components, such as proteins, lipids, and nucleic acids, that mediate remote communication between cells in vivo. EVs can be further classified as exosomes, microvesicles, and apoptotic bodies [21–24]. Exosomes have a diameter of approximately 50–150 nm, have surfaces inlaid with CD81, CD9, and other membrane protein signaling molecules, and are mainly formed by the release of intracellular endosomes through exocytosis. They first form endosomes through endocytosis, and early endosomes then mature into multivesicular bodies, which are transformed mainly in the following two ways: fusion with lysosomes or autophagosomes and subsequent degradation; or fusion with the plasma membrane and then the release as exosomes [25–27]. Exosomes are secreted by almost all cells in an organism, including blood cells (e.g., T lymphocytes, B lymphocytes, and platelets), mesenchymal stem cells, and other cells (e.g., epithelial cells, adipocytes, and astrocytes) [28–30]. Exosomes are present in almost all human bodily fluids, including saliva, breast milk, cerebrospinal fluid, ascitic fluid, urine, and semen [31–33]. They reach target cells through humoral circulation and act in three main ways: direct fusion, endocytosis, and receptor-ligand interaction (Fig. 2). Exosomes are important carriers of signaling components such as proteins, nucleic acids, and lipids, forming a novel cell-cell information transfer system that participates in physiological and pathological processes such as cell proliferation, cell differentiation, cell migration, and intercellular communication [34–36]. By participating in pathophysiological processes, exosomes influence the development of a variety of diseases, such as diabetes, osteoarthritis, neurodegenerative diseases, and tumors [37, 38].

Given that exosomes have been widely studied in disease models, an increasing number of exosome-related databases and online analysis applications have been developed [39–48] (Table 1). For example, the EV microRNA (miRNA) database, which collates and analyzes 462 miRNA expression profile datasets from 17 tissues/diseases, provides multiple functional modules, namely, miRNA expression profiles and information on EV samples from different sources [39]. The ExoCarta database contains information on tissue/cell types for exosome isolation as well as the biophysical and molecular properties of exosomes. Investigators can search for content types (RNA, protein, and lipid), species, tissue/cell types, and gene symbols [40]. The CMEP database contains large-scale circulating miRNA datasets from different platforms that provide miRNA expression profiles, functional pathway enrichment analyses of miRNA target genes, and feature selection methods [41].

A large proportion of these exosome-related databases and online analysis applications can be used to analyze tumor models; however, few can be used to analyze tumor-associated immune cell-derived exosomes. Studies have increasingly reported not only that tumor cell-derived exosomes can act on the immune cells in the TME, but that immune cell-derived exosomes can also act on tumor cells or other immune cells, most of which are TAMs [49]. Tumor cell-derived exosomes affect tumor development by influencing TAMs polarization and phagocytosis [50]. For example, Wolf et al. [51] demonstrated that in metastatic osteosarcoma, ingestion of exosomes derived from K7M3 and DML8 osteosarcoma cell lines into TAMs in the lungs reduced phagocytic activity and the killing effect of TAMs on tumor cells. Ying et al. [52] showed that exosomal miR-222-3p derived from epithelial ovarian cancer (EOC) cells polarizes TAMs-like macrophages into the M2 phenotype through suppression of the cytokine signaling 3 (SOCS3)/signal transducer and activator of transcription 3 (STAT3) signaling axis, accelerating the progression of EOC. Gabrusiewicz et al. [53] reported that monocytes can preferentially and rapidly absorb glioblastoma stem cell-derived exosomes, releasing multiple factors, particularly STAT3—a key molecular hub for tumor-mediated immunosuppression—thereby promoting the expression of programmed death ligand 1 (PD-L1) and polarization into the M2 phenotype.

In contrast, TAMs-derived exosomes have received widespread attention for their role in intercellular information exchange. They participate in different stages of tumor development through the secretion of various bioactive factors. Given that TAMs-derived exosomes are easily accessible and highly stable, they have good prospects as diagnostic biomarkers for tumors and as therapeutic targets. Therefore, through a literature overview, we summarize the potential molecular mechanisms and functions of TAMs-derived exosomes at different stages of tumor development. We also discuss the potential of exosomes as diagnostic biomarkers and therapeutic targets for cancer, as well as the relevant challenges and prospects.

**Table 1:** Exosomes-related databases or tools

Database	Functions	Website	Ref.
EVmiRNA	It provides sample information on miRNA expression profiles and EVs from different sources; miRNAs specifically expressed in different EVs as well as miRNA annotations including miRNA expression in EVs and the cancer genome atlas (TCGA) types; miRNA pathway regulations and miRNA functions.	http://bioinfo.life.hust.edu.cn/EVmiRNA	[39]
ExoCarta	It contains information about the tissue/cell types for isolating exosomes and functional studies of exosomes, including mass spectrometry, western blotting, biological pathways and protein-protein interaction (PPI) networks associated with exosomal proteins. Users can search by content types (RNA, protein, and lipid), species, tissue/cell types and genetic symbols.	http://www.exocarta.org	[40]
CMEP	It contains large-scale circulating miRNA datasets from different platforms, it provides miRNA expression profiles in exosomes, functional pathway enrichment analysis of miRNA target genes and feature selection methods.	http://syslab5.nchu.edu.tw/CMEP	[41]
exoRBase	It is the integration and visualization of RNA expression profiles based on RNA sequencing datas of exosomes derived from human body fluids, containing circRNA, lncRNA and mRNA data profiles, it provides annotations, expression levels and possible original tissues.	http://www.exoRBase.org	[42]
BoMiProt	It is a comprehensive database of bovine milk protein data containing information on exosomal protein function, biochemical properties, and post-translational modifications. In addition, users can search by milk fractions, post-translational modifications, and/or structures by filtering.	http://bomiprot.org	[43]
Xeno-miRNet	The aim is to search and explore heterologous exosomal miRNAs and their potential gene targets in different host species.	http://xeno.mirnet.ca	[44]
NONCODEv5	Construction of human lncRNA-disease and single nucleotide polymorphism-lncRNA-disease relationships; display of human exosomal lncRNA expression profiles; prediction of RNA secondary structures of non-coding human transcripts.	http://www.bioinfo.org/noncode/	[45]
miRandola	Inferring the potential biological functions of circulating miRNAs and their association with phenotypes.	http://atlas.dmi.unict.it/mirandola/index.html	[46]
Vesiclepedia EVpedia	Manual searchable library of molecular data (lipids, RNA, and proteins) for Evs, including apoptotic bodies, exosomes, large dense core vesicles, microparticles and shedding microvesicles, also searchable by species, vesicles, and sample types. It is a community web portal for EVs research, containing data on prokaryotic and eukaryotic EVs.	http://www.microvesicles.org http://evpedia.info	[47] [48]

## Potential molecular mechanisms of TAMs-derived exosomes in tumor development

### TAMs-derived exosomal proteins

TAMs-derived exosomes participate in the development and progression of tumor [54]. In TAMs-derived exosomes, some proteins involved in the positive regulation of inflammation, such as FCER1G, TBK1, and PRKDC, are decreased; however, the levels of cathepsins, such as cathepsin B and S, which promote tumor growth and angiogenesis, are increased. These proteins participate in signal transduction, inflammation, immune response, and tumor migration [55]. Exosomes secreted by M2-type TAMs transmit information between TAMs and gastric cancer (GC) cells. Apolipoprotein E (ApoE) is highly expressed in TAMs-derived exosomes of GC cells, and exosomes secreted by M2-type TAMs transfer ApoE to GC cells, activate the PI3K-Akt signaling pathway and promote cytoskeletal remodeling and GC cell migration [56]. IL-6 is an important member of the cytokine network that mainly transmits signals through the STAT3 signaling pathway in cancer, thereby promoting tumor invasion and metastasis [57]. Yu et al. [58] demonstrated that IL-6 levels were increased in apoptotic cancer cells exposed to macrophage-derived exosomes, thus promoting STAT3 phosphorylation and influencing breast cancer growth and metastasis.

Interestingly, TAMs-derived exosomal proteins can also inhibit tumor progression. A disintegrin and metalloproteinase-15 (ADAM15), a member of the ADAM protein family, inhibits tumor progression [59]. Lee et al. [60] revealed that monocyte-derived macrophages secreted ADAM15, and they injected exosome ADAM15 together with cancer cells into nude mice to construct xenograft mouse models. The results showed that tumor growth in mice was significantly slowed and survival was prolonged. This study suggests that ADAM15 exosomes derived from macrophages participate in tumor inhibition in vivo.

### TAMs-derived exosomal ncRNAs

MiRNAs are a class of small endogenous ncRNAs with an approximate length of 22 nucleotides. They often participate in tumorigenesis, such as invasion, migration, immune escape, and drug resistance, by inhibiting mRNA translation or degradation and downregulating the expression of target genes [61]. TAMs-derived exosomes mediate communication between cancer cells and macrophages through the secretion of miRNAs, which affect tumor proliferation and migration [62]. MiR-21-5p and miR-155-5p are pre-metastatic miRNAs that are highly expressed in TAMs and affect the progression of colorectal cancer. TAMs-derived exosomes down-regulate the expression *BRG1* via miR-21-5p and miR-155-5p, thus promoting the invasion and metastasis of colorectal cancer cells [63]. MiR-223 is a specific miRNA that affects a variety of immune cells, especially macrophages [64]. Yang et al. [65] found that miR-223 secreted by TAMs-derived exosomes is transmitted to breast cancer cells, targeting the Mef2c-β-catenin pathway to promote proliferation, invasion, and metastasis. Treg and Th17 cells are two subtypes of CD4 + T cells that have opposing roles in the immune response [66]. Zhou et al. [67] revealed that STAT3 regulates Treg/Th17 balance in the microenvironment of EOC. TAMs-derived exosomes secrete miR-29a-3p and miR-21-5p, which inhibit the expression of STAT3, lead to Treg/Th17 imbalance, and promote EOC metastasis. N6-methyladenosine (m6A) modifications are associated with cancer development. Methyltransferase-like 14 (METTL14) promotes m6A modification and increases cytoplasmic output [68]. Wang et al. [69] reported that exosomes derived from M1 macrophages can transfer miR-628-5p to hepatocellular carcinoma (HCC) cells and inhibit METTL14 expression, thereby inhibiting HCC progression.

Long non-coding RNAs (lncRNAs) are ncRNAs with more than 200 nucleotides and multiple functions in the nucleus and cytoplasm. They participate in the regulation of chromatin, gene transcription, growth, and differentiation of cancer cells [70–72]. The level of lncMMPA in HCC cells is high, and it can activate the glycolytic pathway in HCC cells [73]. Xu et al. [74] reported that TAMs-derived exosomes delivered lncMMPA to HCC cells and interacted with miR-548 to target ALDH1A3, enhancing aerobic glycolysis and promoting proliferation of HCC cells. In addition, the lncRNA CRNDE is highly expressed in M2 macrophage-derived exosomes of GC and enhances the resistance of GC cells to cisplatin by inhibiting the activation of the PI3K/Akt signaling pathway by PTEN [75]. MiR-26a inhibits esophageal cancer progression; Mi et al. [76] found that TAMs-derived exosomes in esophageal cancer overexpressed the lncRNA AFAP1-AS1, targeting the miR-26a/ATF2 pathway and promoting the proliferation and metastasis of esophageal cancer cells.

Circular RNAs (circRNAs) are novel endogenous ncRNAs. Due to their covalent closed-loop structure, circRNAs are more stable than linear RNAs and have become a topic of interest in recent years [77]. CircRNAs, as competitive endogenous RNAs, often participate in transcriptional regulation and gene expression through spongy miRNAs, and they influence cancer invasion and metastasis [78]. Research has indicated that Circ_0020256 was upregulated in the TAMs-derived exosomes of cholangiocarcinoma and competitively combined with miR-432-5p to target transcription factor E2F3, promoting invasion and metastasis [79]. CircRNA-BTG2 overexpression inhibits proliferation and invasion of glioma cells. Shi et al. [80] found that RBP-J-overexpressing macrophages secrete circRNA-BTG2 from exosomes, which inhibits glioma invasion and proliferation through the miR-25-3p/PTEN pathway. In contrast, when the circRNA-BTG2 is knocked down in exosomes, its inhibitory effect on glioma cell invasion and development is eliminated, indicating that circRNA-BTG2 strongly influences the development and progression of glioma.

### TAMs-derived exosomal metabolites

Exosomes influence metabolic disorders, cancer, and cardiovascular health by facilitating intercellular communication via metabolite transfer. A study [81] found that exosomes mediate communication between adipocytes and macrophages in a spontaneously obese mouse model of leptin-deficient and that retinol-binding protein 4 (RBP4) stimulates macrophage activation and insulin resistance. Obese mice fed a high-fat diet have higher levels of miRNA in the secretions, which may promote insulin resistance in lean mice by downregulating the expression of peroxisome proliferator-activated receptor α (PPARα) in white adipose tissue [82].

Studies have shown that TAMs-derived exosomes are important in the development and progression of many diseases, such as atherosclerosis and Alzheimer’s disease, by influencing lipid metabolism [83]. Lipids carried by exosomes, such as PGE1 and arachidonic acid, can be transferred from macrophages to the TME, and this is closely related to tumor invasion, metastasis, and immunosuppression [84]. Cianciaruso et al. [85] demonstrated that cyclooxygenase-1 (COX1) and thromboxane A synthetase 1 (TBXAS1), which participate in the arachidonic acid pathway, were enriched in TAMs-derived exosomes, but were sparse in the vesicles of MC38 colorectal cancer. The production of thromboxane B2 (TXB2) increased after the transfection of MC38 cells with TAMs-derived exosomes. This study suggests that TAMs-derived exosomes stimulate tumor cells to produce thrombin, thereby promoting tumor cell invasion and metastasis.

## TAMs-derived exosomes and tumor progression

**Fig. 3:**
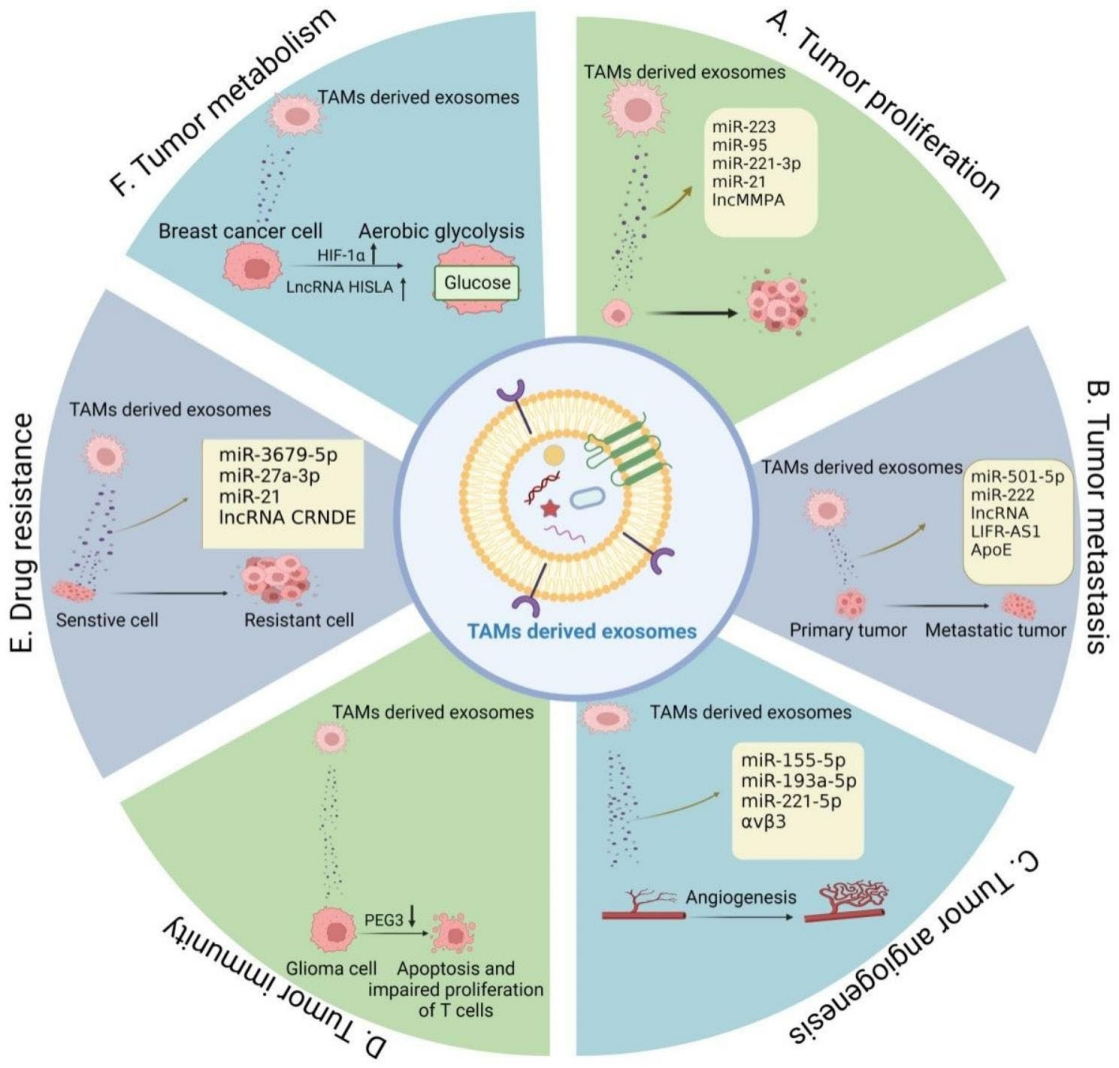
TAMs-derived exosomes can regulate tumorigenesis. **A** TAMs-derived exosomal ncRNAs (e.g., miR-223, miR-95, miR-221-3p, miR-21, and lncMMPA) affect tumor proliferation. **B** TAMs-derived exosomal ncRNAs (e.g., miR-501-5p, miR-222, and lncRNA LIFR-AS1) and proteins (ApoE) affect tumor metastasis. **C** TAMs-derived exosomal ncRNAs (e.g., miR-155-5p, miR-193a-5p, and miR-221-5p) and proteins (αVβ3) affect tumor angiogenesis. **D** TAMs-derived exosomes modulate the immune response to glioma by suppressing PEG3, which further induces apoptosis and impairs T-cell proliferation. **E** TAMs-derived exosomal ncRNAs (e.g., miR-3679-5p, miR-27a-3p, miR-21, and lncRNA CRNDE) affect tumor drug resistance. **F** TAMs-derived exosomal lncRNA HISLA regulates breast cancer metabolism by activating HIF-1α, further promoting cellular glycolysis

Recently, studies have increasingly shown that TAMs-derived exosomes have important roles in tumorigenesis, including malignant proliferation, invasion and metastasis, angiogenesis, immune response, chemotherapy resistance, and tumor metabolism (Fig. 3).

### TAMs-derived exosomes and malignant proliferation of tumors

Malignant proliferation of tumor cells is the most common malignant phenotype during tumor progression [86]. Mutations in tumor suppressor genes can result in a lack of contact inhibition, leading to increased malignant cell proliferation [87]. However, the identification of effective targets to inhibit malignant tumor growth is challenging because of the dearth of related research and clinical experimentation [88, 89].

TAMs-derived exosomes carry various substances into cancer cells, such as lipids, nucleic acids, and proteins, that are closely related to the malignant proliferation of tumors s [90]. Guan et al. [91] showed that the high expression of miR-95 in TAMs-derived exosomes promotes prostate cancer progression. MiR-95 binds to its target gene *JunB* to induce proliferation and epithelial-mesenchymal transformation (EMT) of prostate cancer cells. Low levels of cyclin-dependent kinase inhibitor 1B (CDKN1B) have been associated with EOC. Li et al. [92] found that TAMs-derived exosomes secrete miR-221-3p and inhibit CDKN1B expression, which stimulates EOC proliferation and progression. In contrast, when macrophages were transfected with lentivirus to knock down the expression of miR-221-3p, the proliferation of EOC cells was inhibited and the cell cycle was arrested at the G1/S phase according to flow cytometry. Moreover, miR-21 in TAMs-derived exosomes promotes the proliferation of GC cells and inhibits apoptosis by targeting PDCD4 [93]. These results highlight the importance of information exchange between macrophage exosomes and tumor cells in tumor progression. By targeting the above mentioned molecules in TAMs-derived exosomes, the malignant proliferation of tumor cells can be inhibited.

### TAMs-derived exosomes and tumor metastasis

Metastasis encompasses several sequential stages, namely, local invasion, intravasation, extravasation, and ultimately the establishment of colonies at new sites [94]. Tumor metastasis is influenced by various factors, including the intrinsic characteristics of the tumor and reciprocal interactions between tumor cells and their microenvironment [95, 96]. Tumor dissemination significantly affects patient prognosis and overall survival, primarily contributing to both cancer incidence and mortality [97]. Therefore, investigating the mechanisms underlying tumor metastasis is crucial for the prevention and treatment of cancer.

TAMs-derived exosomes have an important role in tumor metastasis. Yin et al. [90] demonstrated that M2 macrophage-derived exosomes promote pancreatic ductal adenocarcinoma (PDAC) development. Mechanistically, TAMs-derived exosomal miR-501-3p is delivered to PDAC cells, and miR-501-3p targets TGFBR3 to activate TGF-β signaling pathways to promote PDAC cell invasion and migration. NFIA, a member of the nuclear factor I (NFI) family, is closely associated with tumor development. Zhang et al. [98] found that the TAMs-derived exosomal lncRNA LIFR-AS1 can be transferred from macrophages to osteosarcoma cells and further promote the proliferation and invasion of malignant cells through the miR-29a/NFIA axis. Similarly, exosomes derived from M1 macrophages have an important role in tumor invasion and migration. Qi et al. [99] studied the role of M1 macrophage-derived exosomal miR-222 in B-cell lymphoma, observing that miR-222 promoted the apoptosis of bone marrow mesenchymal stem cells (BMSCs) by targeting B-cell lymphoma 2 (Bcl-2). Specifically, M1 macrophages secrete exosomes that deliver miR-222 to the BMSCs, inhibiting the expression of the anti-apoptotic gene *Bcl-2*, thereby significantly reducing the vitality of BMSCs and inhibiting their invasion and migration. These findings provided new targets for treating B-cell lymphoma. Altogether, these studies indicate that therapies targeting these exosomes may reduce the invasive and metastatic ability of tumor cells.

### TAMs-derived exosomes and tumor angiogenesis

Tumor angiogenesis involves a complex sequence of events, including degradation of vascular extracellular matrix, migration and proliferation of ECs, formation of vascular loops through branching, and generation of a new basement membrane. The vascular endothelial growth factor (VEGF)/VEGF receptor (VEGFR) signaling pathway strongly influences tumor growth and angiogenesis [100]. Hypoxic tumor cells release signals, such as VEGF-A, which induce angiogenic responses. These signals bind to VEGFR-2 in adjacent ECs, eliciting vascular formation [101]. At present, drugs targeting the VEGF/VEGFR pathway, including neutralizing antibodies and tyrosine kinase receptor inhibitors, have passed clinical trials. These include bevacizumab, axitinib, pazopanib, and vandetanib [102].

TAMs regulate ECs through exosomes, thus affecting microangiogenesis in the tumor stroma [100]. Angiogenesis in cancer is associated with downregulation of transcription factor E2F2 [103]. In PDAC, M2 macrophage-derived exosomal miR-155-5p and miR-221-5p induce angiogenesis and increase blood vessel density by downregulating endothelium-dependent E2F2 [104]. GATA-binding protein 3 (GATA3) acts as a transcription factor mediating cell differentiation and proliferation [105]. GATA3 is highly expressed in macrophage-derived exosomes in high-grade serous ovarian cancer, enhancing angiogenesis and endometriosis through EMT and epigenetic regulation [106]. These results show that TAMs-derived exosomes are associated with angiogenic factor levels, and targeted regulation of TAMs-derived exosomes may indirectly inhibit tumor angiogenesis. Similarly, studies have shown that TAMs-derived exosomal miR-193a-5p inhibits TIMP2 mRNA translation by targeting the 3′untranslated region (3′UTR), promoting tumor angiogenesis. Further, in vivo experiments have shown that TAMs significantly enhance angiogenesis, tumor proliferation, and metastasis in mice. In contrast, tumor proliferation and angiogenesis were significantly reduced when miR-193a-5p expression was inhibited. High expression levels of TAMs-derived exosomal miR-193a-5p are directly correlated to angiogenesis and tumor deterioration, which lead to poor prognosis in patients with renal cell carcinoma (RCC). This study provides a new approach for anti-vascular therapy for RCC [107].

### TAMs-derived exosomes and immune response

Tumor immunity usually refers to the activation of the immune system when immune cells infiltrate and contact with tumors, as well as the continuous death of tumor cells to release antigens. Then, T and NK cells are activated and antibodies are produced to destroy the tumor cells [108]. The ability of tumors to evade surveillance by the immune system has been considered a barrier to the success of cancer immunotherapy. Additionally, within TME, TAMs are the prevailing inflammatory immune cells. These TAMs secrete various inflammatory factors, chemokines, and cytokines that regulate the immune response of tumors, particularly immune evasion [109]. Therefore, targeting TAMs-derived exosomes may be an effective strategy to overcome tumor immune evasion.

Yang et al. [110] reported that, in glioma, TAMs-derived exosomes suppress CD8^+^ T cell proliferation, immune cell activity, and IFN-γ levels by suppressing PEG3, thereby suppressing glioma cell apoptosis and further promoting glioma cell immune evasion through in vitro and in vivo experiments. Exosomes secreted by M2 macrophages significantly enhance the proliferation and anti-apoptotic ability of colon cancer cells. Moreover, a further study revealed that M2 macrophages carry miRNAs through exosomes to regulate the information exchange between cancer cells. In colon cancer cells, the overexpression of miR-155-5p and IL-6, along with the reduced expression of zinc-finger-type-containing 12B (ZC3H12B), promotes the progression of colon cancer. Among the ZC3H12 family members, ZC3H12B is recognized as the most active member involved in inflammation and mRNA degradation. IL-6 triggers the phosphorylation of STAT3 in colon cancer cells, thereby facilitating their migration. Mechanistically, when miR-155-5p is delivered from M2 macrophages to colon cancer cells via exosomes, it disrupts the ability of ZC3H12B to reduce the stability of IL-6, resulting in immune evasion and tumor formation [111]. Lu et al. [112] found that exosomes derived from TAMs can transfer miR-29a-3p to ovarian cancer cells. Subsequently, a nude mouse xenograft model demonstrated that the depletion of TAM-derived exosomal miR-29a-3p can regulate the FOXO3-AKT/GSK3β axis. This regulation leads to the suppression of PD-L1 expression, ultimately inhibiting the proliferation of ovarian cancer cells and immune evasion. Mechanistically, low expression of FOXO3, which is associated with poor prognosis in ovarian cancer, suppresses GSK3β activity and upregulates PD-L1 expression, thereby inhibiting T-cell activity and ultimately facilitating immune evasion. This study provides experimental evidence supporting the potential therapeutic targeting of miR-29a-3p in ovarian cancer. Moreover, researchers demonstrated that M2 macrophage-derived exosomes may promote GC progression by activating the P38MAPK signaling pathway, simultaneously promoting high PD-L1 expression in GC cells, assisting GC cells in achieving immune evasion, and leading to poor prognosis [113].

### TAMs-derived exosomes and chemotherapy resistance

Chemotherapy is the fundamental treatment for malignant tumors. However, the emergence of drug resistance in tumor cells often results in chemotherapy failure [114]. Chemotherapy resistance encompasses two major categories: intrinsic resistance (existing prior to drug exposure) and acquired resistance (developing after drug exposure). Tumor resistance is intricately associated with a range of factors, including physical parameters (such as size, shape, and volume), genomic alterations, and the TME classification. Recent studies have identified a plethora of tumor cell phenotypes that are closely linked to treatment resistance [115]. Exosomes have emerged as crucial mediators in this ecosystem. Consequently, targeting TAMs-derived exosomes is a promising approach for addressing tumor drug resistance [116].

Yuan et al. [117] examined the role of TAMs-derived exosomes in the sensitivity of lung adenocarcinoma cells to the targeted drug gefitinib. They discovered that M2 macrophage-derived exosomes significantly reduced the apoptosis of HCC827 and PC9 cell lines induced by gefitinib, possibly through activation of the AKT/ERK1/2/STAT3 pathway. This study offers fresh insights into ways to delay or block the development of resistance to epidermal growth factor receptor (EGFR)-targeted drugs. To further study the role of M2 macrophage-derived exosomes in glioblastoma multiforme, Chuang et al. [118] used co-cultured human glioblastoma cell lines (U87MG and LN18) with clinically isolated glioma-associated macrophages (GAMs). They found that compared to the parental control, the colony-forming and tumorigenic capacities were notably enhanced. Through a series of experiments, they discovered that this may be associated with increased expression of Sox2, Oct4, and Nestin, and reduced expression of glial fibrillary acidic protein (GFAP). Specifically, miR-21, which is enriched in GAM-derived exosomes, increases the resistance to temozolomide (TMZ) by modulating the STAT3/miR-21/PDCD4 signaling pathway. MiR-21 levels are positively correlated with the malignancy of glioblastoma. This study also found that the STAT3 inhibitor pacritinib reduced the quantity of GAM-derived exosomes enriched with miR-21, highlighting its potential as an adjuvant to TMZ therapy. Wang et al. [119] demonstrated that M2 macrophage-derived exosomes strongly promote cisplatin resistance in lung cancer both in vitro and in vivo. MiR-3679-5p can be transferred from M2 macrophages to lung cancer cells via the internalization of exosomes, which induces chemical resistance to cisplatin in lung cancer cells through the miR-3679-5p/NEDD4L/c-Myc axis. Specifically, miR-3679-5p inhibits the transcription of NEDD4L, which mediates the lysosomal degradation of target proteins and is downregulated in various types of cancer to promote cancer progression. It also regulates c-Myc stability via ubiquitination. Stable c-Myc promotes aerobic glycolysis in tumors and induces chemical resistance to cisplatin. The discovery of this target provides a scientific basis for lung cancer management. Osimertinib is an important first-line treatment for *EGFR* mutation-positive non-small-cell lung cancer (NSCLC). Through nude mouse tumorigenicity experiments, Wan et al. [120] observed that M2-type TAMs-derived exosomes highly expressing MSTRG.292666.16 enhanced the resistance of NSCLC to osimertinib. Mechanistically, M2-type TAMs-derived exosomes promoted the resistance of NSCLC to osimertinib by regulating the MSTRG.292666.16/miR-6836-5p/MAPK8IP3 pathway. Abnormal activation of the MAPK signaling pathway may cause loss of cell differentiation and apoptosis, promoting the resistance of lung cancer cells to osimertinib. The discovery of this pathway may have value in the management of osimertinib-resistant NSCLC. Furthermore, Liu et al. [121] demonstrated that M2 macrophage-derived exosomes blocked the propranolol-induced decrease in proliferation and increase in apoptosis of infantile hemangioma cells. Using a xenograft mouse model, they found that mice pre-treated with M2 macrophage-derived exosomes exhibited enhanced propranolol resistance and significantly increased hemangioma volume and weight. Specifically, M2 macrophage-derived exosomes deliver miR-27a-3p to hemangioma stem cells, reducing their sensitivity to propranolol by targeting dickkopf-related protein 2 (DKK2).

### TAMs-derived exosomes and metabolic reprogramming of tumors

Metabolic reprogramming refers to a cellular mechanism that alters metabolic patterns to facilitate cell proliferation and growth in response to energy demand. This process confers resilience to external stressors and novel functionalities to cells [122]. During rapid proliferation, tumor cells undergo metabolic reprogramming due to an increased demand for energy [123]. Tumor cells primarily rely on glycolysis for their energy metabolism. Although oxidative phosphorylation is more efficient in generating ATP per glucose molecule, glycolysis has a significantly higher rate of ATP production. Consequently, tumor cells prioritize glycolysis for ATP generation as their primary energy source irrespective of oxygen availability [124, 125]. There is a significant dearth of effective drugs that specifically target the glycolytic pathway in tumors. Identifying potential molecular targets associated with tumor glycolysis is a promising strategy to impede tumor growth and regulate tumor progression.

Numerous studies have recently highlighted the impact of TAMs-derived exosomes on tumor progression by regulating tumor cell glycolysis. Specifically, Chen et al. [126] discovered that TAMs enhance aerobic glycolysis and confer resistance to apoptosis in breast cancer cells by delivering the lncRNA HISLA through exosomes. Specifically, HISLA inhibits the hydroxylation and degradation of HIF-1α by decreasing the interaction of PHD2 with HIF-1α. The HIF-1α is a central regulator of hypoxia-induced genes and repair of the intracellular oxygen environment, which determines the fate of intracellular glucose, namely, oxidation or glycolysis. Conversely, inhibiting the EV-mediated delivery of HISLA suppresses glycolysis and chemoresistance in breast cancer. Furthermore, Zhang et al. [127] revealed that TAMs act on glioma cells through exosomal secretion of IL-6 and promote PDPK1-mediated phosphoglycerate kinase 1 (PGK1) T243 phosphorylation in glioma cells. PGK1 is a cytoplasmic glycolytic enzyme that is overexpressed in many cancers. The PGK1 T243 phosphorylation alters the PGK1 and substrate affinity, promoting aerobic glycolysis and tumor growth in glioma cells. This study suggested that blocking PGK1 T243 phosphorylation may be a potential therapeutic target to inhibit aerobic glycolysis and tumor growth in glioma cells.

## Clinical value of TAMs-derived exosomes in cancers

### TAMs-derived exosomes as novel potential diagnostic and prognostic biomarkers of cancers

Liquid biopsy is a new concept that has emerged in recent years, unlike traditional tissue biopsy, which uses invasive means to obtain lesion specimens. Liquid biopsy is a time-efficient and minimally invasive method to obtain part of the patient’s body fluid components (e.g., blood, saliva, and urine) through noninvasive or minimally invasive means. Moreover, through molecular biology methods such as high-throughput omics technologies, it detects free circulating tumor DNA fragments, exosomes, or circulating tumor cell components. It is essential in tumor drug resistance detection, prognosis assessment, and early tumor screening. It helps open a new era of precision medicine [128].

Exosomes can carry RNA, DNA, proteins, lipids, and metabolites, and are widely found in all human body fluids, including saliva, cerebrospinal fluid, breast milk, ascites, semen, and urine [129–131]. Recently, exosomes, as a hot new research focus area, have been widely used in liquid biopsy. Due to the characteristics of large information content, easy enrichment, high detection rate, and better stability [132, 133], exosomes may be served as potential diagnostic and prognostic biomarkers, and therapeutic targets for the monitoring, detection and therapy of cancers [134].

TAMs-derived exosomes exhibit promising potential as molecular biomarkers for noninvasive testing, providing novel liquid biopsy indicators for early tumor diagnosis and prognostic assessment [104]. Recent research has revealed a significant correlation between the dysregulated levels of TAMs-derived exosomes (ncRNAs and proteins) and the clinicopathological characteristics of patients with cancer. These characteristics include Tumor-Node-Metastasis (TNM) staging, angiogenesis, and overall prognosis [79, 90, 98, 104, 107, 112, 118, 135–137] (Table 2). Yin et al. [90] found significant elevation in exosomal miR-501-3p expression in patients with metastatic PDAC compared to those without metastasis. This elevated expression strongly correlates with tumor lymph node metastasis and angiogenesis. Thus, exosomal miR-501-3p is a promising biomarker for predicting the invasiveness and metastatic potential of PDAC. Moreover, its expression levels have potential as a valuable biomarker for assessing and monitoring therapeutic responses in patients with metastatic PDAC. Furthermore, similar studies exploring the potential value of TAM-derived exosomes as biomarkers have been conducted. For example, Zhang et al. [98] demonstrated that upregulation of lncRNA LIFR-AS1 in osteosarcoma cells significantly increases cell proliferation and invasion. Further investigation revealed that exosomes facilitate the transfer of LIFR-AS1 from macrophages to osteosarcoma cells. The identification of exosomal lncRNA LIFR-AS1 as a potential biomarker for evaluating osteosarcoma metastasis offers new possibilities for therapeutic approaches in the treatment of this aggressive tumor. In addition, High levels of integrin αVβ3 have been observed in TAMs-derived exosomes from patients with metastatic NSCLC. Its overexpression correlates with tumor vascular infiltration, lung metastasis, and TNM staging [135]. Despite being in the nascent stages of research, increasing evidence suggests the potential of TAMs-derived exosomes as promising cancer biomarkers.

**Table 2:** Correlation between TAMs-derived exosomes and clinicopathological features of cancers

Types of cancers	TAMs-derived exosomes	Expression	Relationship with clinicopathologic features	Ref.
Pancreatic ductal adenocarcinoma	miR-501-3p	↑	TNM stage, tumor angiogenesis, poor prognosis	[90]
	miR-155-5p and miR-221-5p	↑	TNM stage, tumor angiogenesis	[104]
Renal cell carcinoma	miR-193a-5p	↑	TNM stage, poor prognosis	[107]
Ovarian cancer	miR-29a-3p	↑	Poor prognosis	[112]
Glioblastoma	miR-21	↑	TNM stage	[118]
Oral squamous cell carcinoma	miR-31-5p	↑	TNM stage, poor prognosis	[136]
Intracranial aneurysm	miR-155-5p	↑	TNM stage	[137]
Osteosarcoma	lncRNA LIFR-AS1	↑	Poor prognosis, lung metastasis	[98]
Cholangiocarcinoma	Circ_0020256	↑	TNM stage, lymph node metastasis	[79]
Non-small cell lung cancer	αVβ3	↑	TNM stage, poor prognosis	[135]

### TAMs-derived exosomes as novel potential targets of cancer therapy

Immunotherapy encompasses strategies aimed at enhancing the ability of the immune system to combat various diseases, including cancer, with the ultimate goal of achieving therapeutic benefits [138]. Increasing evidence highlights the significant role of TAMs in tumor immunotherapy, making TAMs-targeted approaches a promising avenue for innovative cancer treatment [139, 140]. At present, macrophage-targeted therapeutic strategies mainly divide into three aspects [141]: (1) Depletion of existing macrophages in the TME; (2) Reduces the recruitment of macrophages in TME; (3) Repolarization of the M2 macrophages to M1 macrophages in the TME. Recent research has identified several potential therapeutic targets associated with TAMs. Notable targets that have demonstrated promising clinical advancements include CSF-1R [142], CD47 [143], and macrophage chemoattractant factors. Clinical trials have already been conducted for CCR5 inhibitors, such as maraviroc and vicriviroc as well as the dual CCR2/CCR5 antagonist BMS-813,160 [144]. Notably, Singh et al. [145] demonstrated that maraviroc effectively reduces CCR5-mediated proliferation and invasion. In a rat xenograft model, maraviroc showed significant efficacy in reducing liver metastasis of pancreatic cancer. These compelling results suggest that maraviroc is a potentially effective therapeutic option for patients with pancreatic cancer liver metastasis. Another study found that JNJ-40,346,527, a CSF-1R inhibitor, effectively reduces the expression of multiple genes related to EMT, stem cell markers, and resistance mechanisms in diverse CSF-1R-positive lung cancer cell lines (e.g., A549, NCI-H1299, NCI-H157, CALU-1, NCI-H1975, NCI-H358 and NCI-H4660) [146–148]. Additionally, sitravatinib modulates immune checkpoint inhibition by inducing alterations in innate and adaptive immune cells in TME. It enhances the immune response by modulating the ratio of M1 to M2 macrophages, increasing the population of immune-stimulatory M1 macrophages, and concurrently decreasing the presence of immune-suppressive M2 macrophages. The clinical trial (NCT03575598) assessing the neoadjuvant therapy of sitravatinib and nivolumab in oral cancer demonstrated that sitravatinib induced the transition of TAMs from a polarized state to an immunostimulatory phenotype. The TME experienced a reduction in MDSCs, accompanied by an increase in the proportion of M1 macrophages and a decrease in the proportion of M2 macrophages [149]. However, targeted immunotherapy against TAMs still faces significant challenges, primarily because of the limited number of clinical trials conducted. These challenges can be attributed to the heterogeneous population of monocytes and the extensive array of chemokines involved in targeting their ligands and receptors within tumors.

In recent years, exosomes have gained attention as “natural nanospheres” for drug delivery applications. Exosomes exhibit superior cellular uptake compared to liposomes, resulting in the efficient delivery of functional cargo upon injection into the body. Moreover, exosomes have minimal immune clearance rates, particularly when administered to mice, making them ideal for targeted drug delivery [141]. Additionally, exosomes possess excellent tolerability, as evidenced by studies conducted by Kordelas et al. [150], where repeated administration of mesenchymal stem cell-derived exosomes to graft-versus-host disease patients showed no toxicity, good tolerability, and minimal side effects. Exosome engineering is a rapid and versatile approach that confers novel characteristics to exosomes, enabling their use as efficient drug delivery vehicles with precise cellular targeting capabilities. One noteworthy example involves the engineering of exosomes to express a fusion protein consisting of an αγ integrin-specific iRGD peptide (CRGDKGPDC) and LAMP-2B. These modified exosomes exhibited selective delivery of KRAS siRNA to αvβ3 integrin-expressing NSCLC cells. The engineered exosomes effectively downregulated the KRAS gene, inhibited tumor growth, and exhibited minimal toxicity [151]. Haney et al. [152] developed a novel delivery system using macrophage-derived exosomes, for the treatment of Parkinson’s disease. This system involves the use of catalase-loaded exosomes. The exosomes were labeled with the lipophilic fluorescent dye DIL to track their uptake by the target neuronal cells (PC12 cells). This study demonstrated efficient internalization of macrophage-derived exosomes by neurons, resulting in their accumulation on the neuronal cell membrane. Importantly, catalase-loaded exosomes demonstrate neuroprotective effects against oxidative stress.

**Fig. 4:**
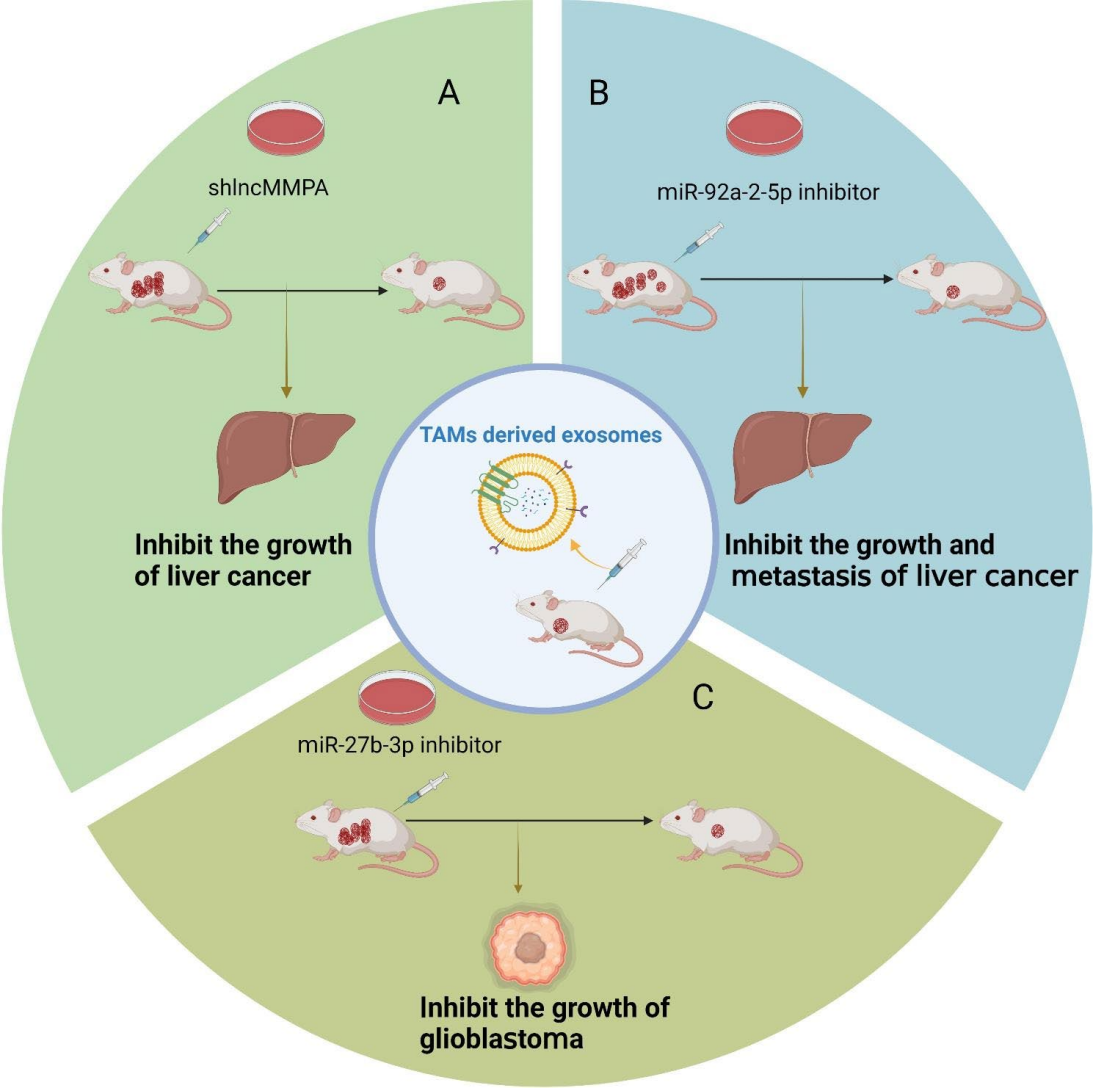
The potential application of TAMs-derived exosomes in the targeted therapy of cancer. In a mouse xenograft model, TAMs-derived exosomal shlncMMPA suppresses the growth of HCC (**A**); TAMs-derived exosomal miR-92a-2-5p inhibitor can suppress the growth and metastasis of HCC (**B**); TAMs-derived exosomal miR-27b-3p inhibitor has a decelerating effect on glioblastoma cells growth (**C**)

Accumulating evidence suggests that TAMs-derived exosomes have the potential to predict the prognosis of cancer patients and identify tumor treatment targets. However, further studies are required to effectively apply these findings in clinical practice. Research on the involvement of TAMs-derived exosomes in tumor development is primarily at the experimental stage and relies on animal models (Fig. 4). For example, in a liver cancer xenograft model established by subcutaneously injecting Hep3B cells into 4-week-old male BALB/c nude mice, the injection of TAMs-derived exosomes containing lncMMPA increased glucose consumption and significantly promoted tumor growth. Conversely, inhibition of exosome production using GW4869 resulted in a significant decrease in glucose consumption and cell proliferation in mice [74]. In a mouse model of liver cancer, abnormal expression of exosomal miR-92a-2-5p inhibited the translation of the androgen receptor, leading to alterations in the PHLPP/p-AKT/β-catenin signaling pathway and increased invasiveness of liver cancer cells. Preclinical studies have shown that inhibiting miR-92a-2-5p can partially restore androgen receptor expression and impede tumor growth [153]. Zhao et al. [154] used a mouse xenograft model to investigate the function of M2 macrophages-derived exosomes in glioblastoma. The findings demonstrated that M2-TAMs-derived exosomes containing miR-27b-3p facilitate tumor progression by activating the MLL4/PRDM1/IL-33 axis. Conversely, the administration of miR-27b-3p inhibitor in M2-TAMs-derived exosomes resulted in a significant increase the survival rate of mice.

## Discussion and future perspectives

TME is a crucial site for targeted cancer treatment strategies and immunotherapy. Recent advances in single-cell sequencing and spatial transcriptomic technologies have enabled the identification of a myriad of immune cell subtypes in the TME that are intricately involved in tumor therapy. TAMs have received considerable attention for their immense therapeutic potential in various types of tumors, particularly solid tumors [141, 155]. TAMs-derived exosomes have emerged as key players in establishing communication between TAMs and the TME and mediating bidirectional signaling within the TME. These exosomes contain diverse bioactive constituents, including lipids, ncRNAs, and proteins, which can influence tumor progression [156, 157]. Hence, further investigation of the effects and mechanisms of TAMs-derived exosomes on tumor development, along with their clinical implications as therapeutic targets, may reveal the crucial role of TAMs in the TME.

Numerous studies have reported the pivotal role of TAMs-derived exosomes in various stages of tumor development. These exosomes represent potential targets for cancer treatment and serve as novel liquid biopsy markers for early tumor diagnosis and patient monitoring [158]. The unique characteristics of exosomes contribute to their significant involvement in tumor development. Firstly, exosomes are readily available in multiple body fluids, including blood, breast milk, amniotic fluid, urine, and saliva [159]. Secondly, exosomes exhibit high stability because of their lipid bilayer structure. This stability allows them to protect cargo molecules, such as hydrophobic or hydrophilic drugs, and facilitates their specific binding to receptors on target cells. Thirdly, exosomes, particularly TAMs-derived exosomes, possess tissue-like characteristics. These exosomes encapsulate RNA, proteins, metabolites, and other cargo molecules that resemble those present in cancer cells, enabling the monitoring of dynamic alterations in tumor progression. This has led to an increased focus on identifying cancer-specific exosomes that can serve as diagnostic biomarkers [56, 69, 75]. The advantages of exosomes have led to a notable increase in research in this field, making them a prominent area of investigation.

However, practical implementation of TAMs-derived exosomes in clinical settings requires significant progress beyond scientific research. The study of TAMs-derived exosomes has encountered several challenges and limitations in recent years, including the absence of optimized protocols for efficient exosome isolation and purification, inadequate production yield, and lack of a clear understanding of the specific mechanisms involved. Currently, the common techniques for isolating and purifying EVs include ultracentrifugation, filtration, precipitation, and immune enrichment. However, ultracentrifugation, considered the gold standard, is time-consuming and technically complicated. Moreover, these purification techniques often face difficulties in differentiating exosomes from other EVs, further complicating the research process [160, 161]. Additionally, although an increasing number of research teams are dedicated to the development of in vitro-engineered exosomes, the application of exosome-based therapeutics is still in its early stages. The current stage is characterized by immature technologies and equipment, posing challenges in estimating the costs associated with large-scale production [162]. Although the therapeutic potential of TAMs-derived exosomes and their components (such as miRNA, lncRNA, circRNA, and proteins) has been demonstrated in various animal models and progress has been made in the targeted modification of exosomes in experimental settings, the intricate microenvironment within the human body and the precise role of TAMs in tumor ecosystems have yet to be fully elucidated. Consequently, substantial uncertainty remains regarding the efficacy of therapies targeting TAMs or TAMs-derived exosomes. Further clinical experiments and trials are necessary to determine whether modified exosomes can retain their desired therapeutic effects upon administration [22].

In conclusion, this review article has analyzed the function and underlying mechanisms of TAMs-derived exosomes as mediators of intercellular communication between TAMs and the TME. Their involvement in critical processes, such as tumor cell proliferation, invasion, metastasis, angiogenesis, immune responses, drug resistance, and tumor metabolism has shed light on their significant role in the pathogenesis of cancer. Additionally, the clinical significance of TAMs-derived exosomes as potential biomarkers for liquid biopsy of cancer has been highlighted. Although our current understanding of the precise mechanisms and functions of TAMs-derived exosomes is incomplete, there is a gradual revelation of the “mysterious veil” surrounding the TME. In the near future, TAMs-derived exosomes are expected to demonstrate remarkable clinical translational benefits as diagnostic biomarkers and therapeutic targets in cancer.

## Data Availability

Not applicable.

## Abbreviations

TME: Tumor microenvironment
TAMs: Tumor-associated macrophages
EVs: Extracellular vesicles
ncRNAs: Non-coding RNAs
IL: Interleukin
CAFs: Cancer-associated fibroblasts
ECs: Endothelial cells
DCs: Dendritic cells
NK: Natural killer
MDSCs: Myeloid-derived suppressor cells
miRNA: microRNA
EOC: Epithelial ovarian cancer
SOCS3: Cytokine signaling 3
STAT3: Signal transducer and activator of transcription 3
PD-L1: Programmed death ligand 1
GC: Gastric cancer
ADAM15: A disintegrin and metalloproteinase-15
m6A: N6-methyladenosine
METTL14: Methyltransferase-like 14
HCC: Hepatocellular carcinoma
lncRNAs: Long non-coding RNAs
circRNAs: Circular RNAs
RBP4: Retinol-binding protein 4
PPARα: Peroxisome proliferator-activated receptorα
COX1: Cyclooxygenase-1
TBXAS1: Thromboxane A synthetase 1
TXB2: Thromboxane B2
EMT: Epithelial-mesenchymal transformation
CDKN1B: Cyclin-dependent kinase inhibitor 1B
PDAC: Pancreatic ductal adenocarcinoma
NF1: Nuclear factor I
BMSCs: Bone marrow mesenchymal stem cells
Bcl-2: B-cell lymphoma 2
VEGF: Vascular endothelial growth factor
VEGFR: VEGF receptor
GATA3: GATA-binding protein 3
RCC: Renal cell carcinoma
ZC3H12B: Zinc-finger-type-containing 12B
EGFR: Epidermal growth factor receptor
GAMs: Glioma-associated macrophages
GFAP: Glial fibrillary acidic protein
TMZ: Temozolomide
NSCLC: Non-small-cell lung cancer
DKK2: Dickkopf-related protein 2
PGK1: PDPK1-mediated phosphoglycerate kinase 1
TNM: Tumor-Node-Metastasis
